# A Novel Bunyavirus-Like Virus of Trypanosomatid Protist Parasites

**DOI:** 10.1128/genomeA.00715-16

**Published:** 2016-08-04

**Authors:** Natalia S. Akopyants, Lon-Fye Lye, Deborah E. Dobson, Julius Lukeš, Stephen M. Beverley

**Affiliations:** a Department of Molecular Microbiology, Washington University School of Medicine, St. Louis, Missouri, USA; b Institute of Parasitology, Biology Centre, and Faculty of Sciences, University of South Bohemia, Ĉeské Budějovice, Czech Republic; c Canadian Institute for Advanced Research, Toronto, Ontario, Canada

## Abstract

We report here the sequences for all three segments of a novel RNA virus (LepmorLBV1) from the insect trypanosomatid parasite *Leptomonas moramango*. This virus belongs to a newly discovered group of bunyavirus-like elements termed Leishbunyaviruses (LBV), the first discovered from protists related to arboviruses infecting humans.

## GENOME ANNOUNCEMENT

*Bunyaviridae* comprise >350 species, often arthropod-borne and including serious human, animal, and plant pathogens (1). In searching for novel viruses from trypanosomatid protists (Kinetoplastida, supergroup Excavata), we found a new viral element within the insect parasite *Leptomonas moramango* (2).

RNA was prepared from an uncloned strain of parasites (2) using TRIzol reagent (Thermo Fisher), treatment with DNase I (Thermo Fisher), and purified with RNA Clean & Concentrator-25 (Zymo Research). Replicative viral double-stranded RNAs (dsRNAs) were visualized following treatment with S1 nuclease (Thermo Fisher), separation by agarose gel electrophoresis, and staining by ethidium bromide (3). Three prominent bands with sizes of about 6, 1.3, and 0.7 kb were observed (L, M, and S, respectively). Total RNA was depleted of rRNA with the Ribo-Zero kit (Illumina), fragmented to 200 to 600 nucleotides (nt), and used as a template for the generation of multiplexed TruSeq cDNA libraries. These were sequenced (2 × 101 cycles, paired-end reads) on the HiSeq 2500 (Illumina). A total of 45,411,994 reads were obtained, of which 722,520 (1.6%) were assigned to viral segments (CLC Genomics Workbench).

For each segment, we identified two related similarly sized contigs (L, 5,982 and 6,029 nt; M, 1,205 and 1,154 nt; S, 662 and 820 nt). Within each pair, one was more abundant, as judged by read depth coverage (L, 1,634 or 471 nt; M, 12,453 or 225 nt; S, 53,243 or 622 nt); we arbitrarily grouped the more abundant segments as “a” and refer to the viruses as LepmorLBV1a and 1b. The G+C compositions of LepmorLBV1s were similar to each other and to that of the meta-transcriptomic assembly (38 to 41%). We were able to detect 9-nt “panhandle” repeats (4, 5) on 6/12 termini within the metatranscriptomic contigs.

Comparisons between the LepmorLBV1a/b segment open reading frames (ORFs) showed significant nucleotide and amino acid identity (52 to 54% and 37 to 50%, respectively). L segments displayed a single ORF encoding 1,979 or 1,982 amino acids (aa), bearing motifs found typically in bunyavirus RNA-dependent RNA polymerases (RdRp) (6, 7). M segments encoded a single ORF of 322 or 321 aa; no database hits were found, but the protein(s) were predicted to have a hydrophobic N-terminal signal sequence. S segments predicted proteins of 165 and 168 aa, which showed structural similarity to the nucleocapsid of other bunyaviruses (4, 5, 8).

Phylogenetic analysis of the RdRp domain showed that LepmorLBV1s grouped together with bunyaviruses, relative to other viral outgroups, but as a clearly distinct clade well separated from *Phlebovirus* or other genera. Additional affinities to *Bunyaviridae* include a negative single-stranded RNA genome, terminal repeated panhandle sequences important for replication and transcription, and three segments typically encoding the viral RdRp, envelope glycoproteins, and the nucleocapsid (4, 5, 7). The *L. moramango* virus thus resembles a group of related viruses discovered recently in the closely related human parasite *Leishmania*, which we have termed *Leishbunyavirus* (LBV).

*L. moramango* may bear cytoplasmic virus-like particles (2) that could correspond to LepmorLBV1. Future studies will establish the evolutionary distribution of LBVs and their potential role in host pathogenicity (9).

### Accession number(s).

The sequences of all segments of LepmorLBV1a and 1b were deposited in GenBank under the accession numbers KX280012 to KX280017.
